# Inveterate Section of Deep Flexor in Pediatric Hand, Periosteum Clues

**DOI:** 10.7759/cureus.96300

**Published:** 2025-11-07

**Authors:** David Mayorga-Naranjo, María Ángeles Blasco-Molla, Carmen Garcia-Espert

**Affiliations:** 1 Orthopaedic Surgery and Traumatology, Hospital Universitari i Politècnic La Fe, Valencia, ESP

**Keywords:** chronic rupture, pediatric hand, periosteum injury, tendon ruputre, ultrasound

## Abstract

Diagnostic delay in flexor tendon injuries is common in pediatric patients, and age influences both recovery and postoperative management. We report the case of a three-year-old girl who presented with the inability to flex the distal interphalangeal joint of the left third finger, two months after the digit became entrapped in a chair. Ultrasonography revealed cortical ossification of the middle phalanx compressing the flexor tendon, without clear evidence of discontinuity. Surgical exploration was undertaken due to a strong clinical suspicion of flexor digitorum profundus rupture, revealing an old laceration with fibrotic changes. The proximal tendon stump was reattached to the distal phalanx using a pull-out suture; the bone lesion was left untreated. At six weeks, radiographs demonstrated ossification consolidation, consistent with periosteal injury secondary to a middle phalanx fracture. This case highlights the importance of examination under sedation for pediatric hand injuries. Periosteal injury can result in callus formation, and after tendon repair, postoperative immobilization may need to be prolonged compared with adult patients.

## Introduction

Flexor tendon injuries of the hand in the pediatric population present unique diagnostic and therapeutic challenges. Their apparently lower frequency contributes to limited clinical experience, but the main difficulties arise from the anatomical and behavioral characteristics of children [1,2]. Although pediatric hand trauma has traditionally been considered relatively benign, a substantial proportion involves deep structures-tendons, nerves, or vessels-potentially leading to significant functional impairment if not managed appropriately [3,4].

In adults, the management of flexor tendon injuries has been extensively studied, with robust evidence supporting controlled early mobilization protocols that aim to balance adhesion prevention against the risk of repair rupture [5,6]. However, these approaches are not always directly applicable to children. The smaller caliber of tendons, limited cooperation during rehabilitation, and differing biological responses necessitate tailored surgical and postoperative strategies [2,7].

While primary repair in the acute phase generally yields favorable outcomes, the prognosis and management of delayed or neglected injuries in children are far less understood [8]. In adults, the traditional time limit for primary repair has been increasingly questioned, with some studies demonstrating acceptable results even beyond three months post-injury in selected cases [9]. In the pediatric setting, however, evidence regarding delayed primary repair is largely anecdotal, underscoring the need for well-documented clinical reports.

Rehabilitation adds another layer of complexity. Some authors have suggested that complete immobilization for up to three weeks may not compromise function in children, possibly due to their greater regenerative potential, enhanced vascularity, and superior scar remodeling [10,11]. Nevertheless, adhesion formation and joint stiffness remain significant concerns, prompting the exploration of modified controlled-motion protocols that have shown promising outcomes [12,13].

Finally, pediatric patients may exhibit unique biological responses to trauma, such as periosteal reactions or localized bone growth, which can influence tendon integrity and digital biomechanics-particularly when diagnosis is delayed [14,15].

In this context, the present report aims to contribute clinical insight through the description of a chronic flexor digitorum profundus tendon laceration in a pediatric hand, accompanied by a critical review of the current literature. We discuss surgical decision-making, rehabilitation considerations, and prognostic factors specific to this underrepresented patient population.

## Case presentation

**Figure 1:**
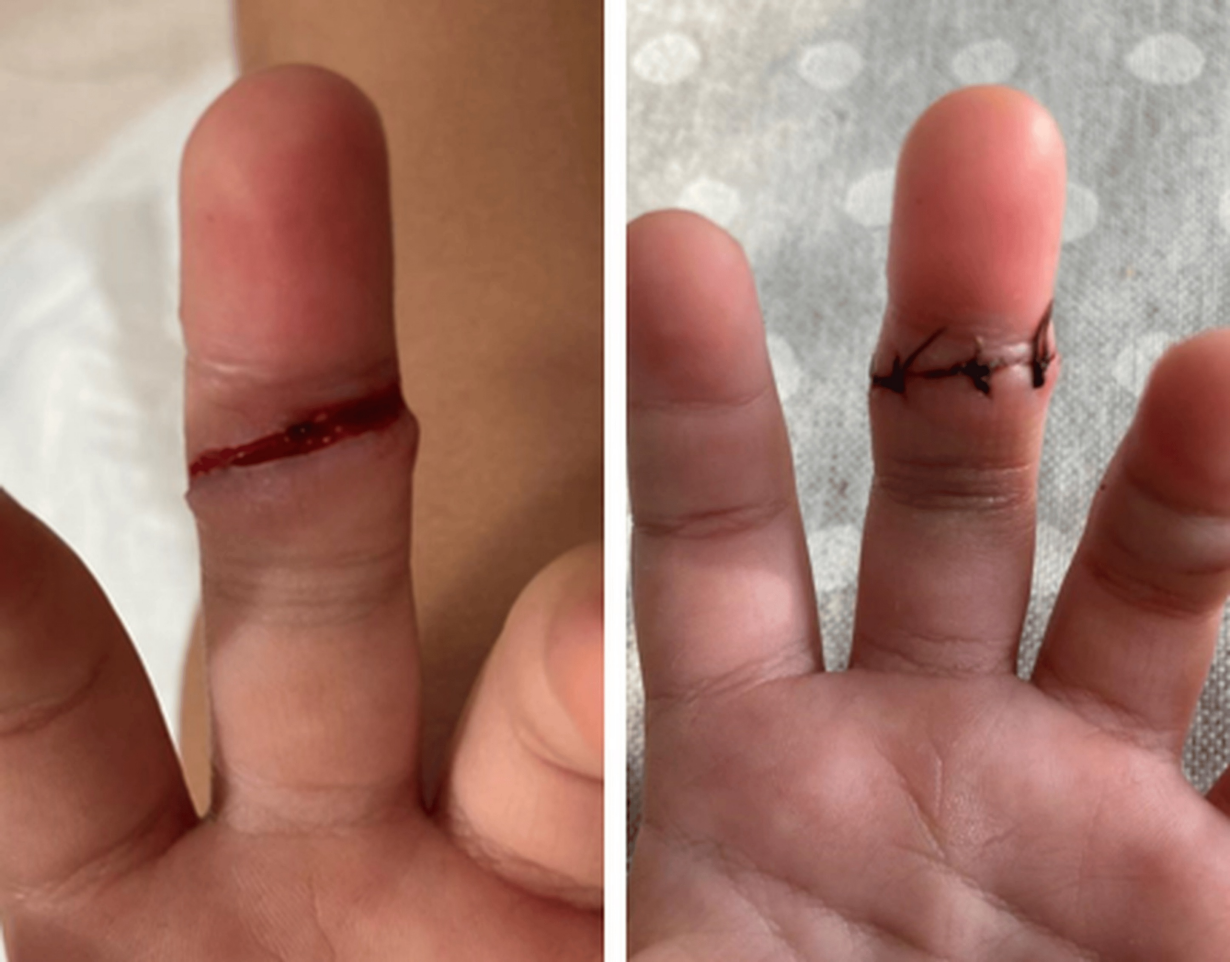
Clinical images on the day of injury Left: incised wound on the volar aspect of the middle phalanx of the left third finger prior to skin suturing; Right: after skin suturing.

We report the case of a three-year-old girl who presented to our hospital with inability to flex the distal interphalangeal joint of the left third finger. Her past medical history included an injury sustained two months earlier, when the digit became entrapped in a folding camping chair, resulting in an incised-contused wound on the volar aspect of Verdan zone II. At that time, she was taken to a nearby primary care center, where skin suturing was performed without exploration for possible tendon involvement, and no imaging studies were obtained (Figure 1).

**Figure 2:**
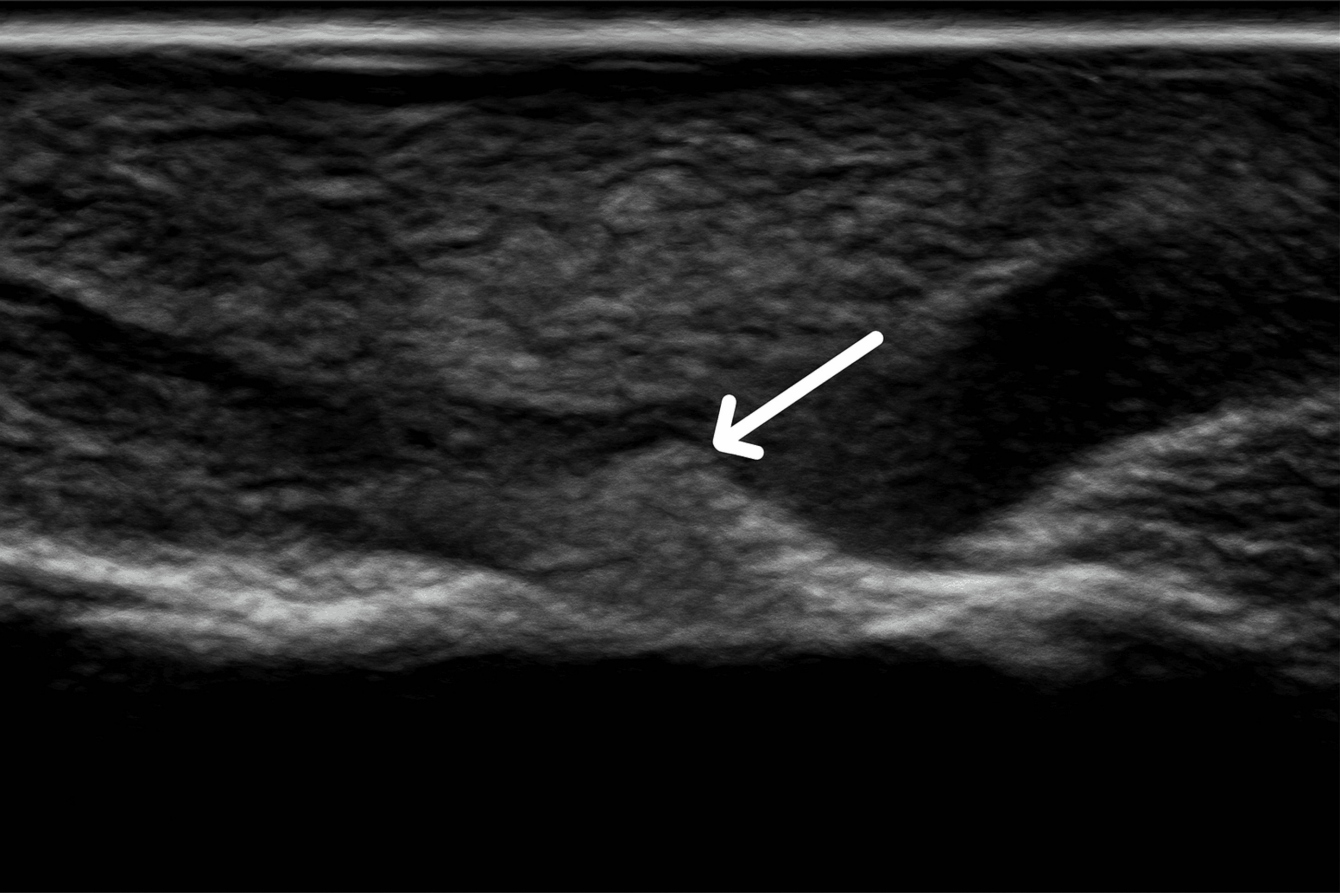
Image consistent with ossification in the middle phalanx, exerting an impression on the flexor tendon

**Figure 3:**
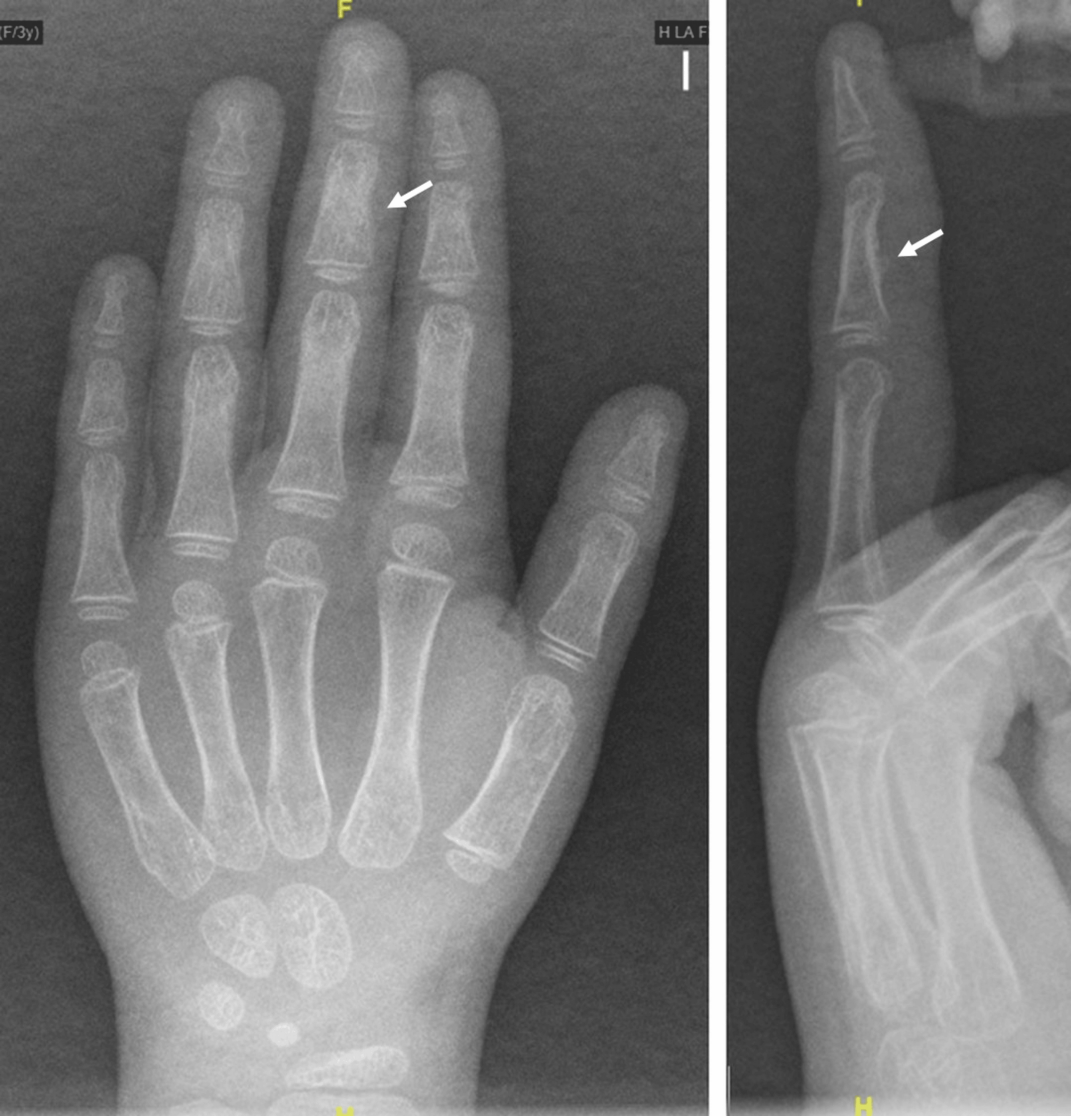
Ossification of the middle phalanx observed on plain radiographs of the hand Left: anteroposterior view; Right: lateral view.

On presentation to our center, there was clinical suspicion of a chronic laceration of the flexor digitorum profundus tendon. Ultrasound examination of the affected finger (Figure 2) revealed a small ossified focus in the mid-volar cortex of the middle phalanx, producing indentation of the flexor tendon and thinning of its distal fibers. No discontinuity or tendon stumps were identified. Plain radiographs of the hand in anteroposterior and lateral views confirmed the presence of cortical ossification in the middle phalanx (Figure 3). Ultrasound was performed to obtain a dynamic, high-resolution evaluation of tendon integrity and to delineate the relationship between the ossified lesion and adjacent soft tissues. Considering the patient’s age and a history of minor trauma several months earlier, the differential diagnosis included post-traumatic calcification, early myositis ossificans, and benign reactive or developmental cortical ossification, whereas neoplastic etiologies were deemed unlikely.

Intraoperatively, a chronic rupture of the flexor digitorum profundus tendon was identified, with fibrotic tissue at the A4 pulley in Verdan zone one. Both edges of the A4 pulley and the fibrotic area around the tendon were carefully dissected. The proximal stump of the flexor digitorum profundus was mobilized and anchored to the distal phalanx using a simple pull-out suture technique. Intraoperative inspection revealed frayed but viable tendon ends with good vascularity, and a gap of approximately 5 mm between the stumps after debridement. The surrounding tissue exhibited mild fibrosis but adequate quality to support tension-free repair.

Postoperatively, the patient was immobilized with a brachial splint, keeping the elbow and metacarpophalangeal joints flexed at 90° and the wrist in 30° of volar flexion, while allowing finger flexion at the interphalangeal joints. At three weeks, the splint was replaced by another brachial splint of similar configuration, but with the wrist in a neutral position.

**Figure 4:**
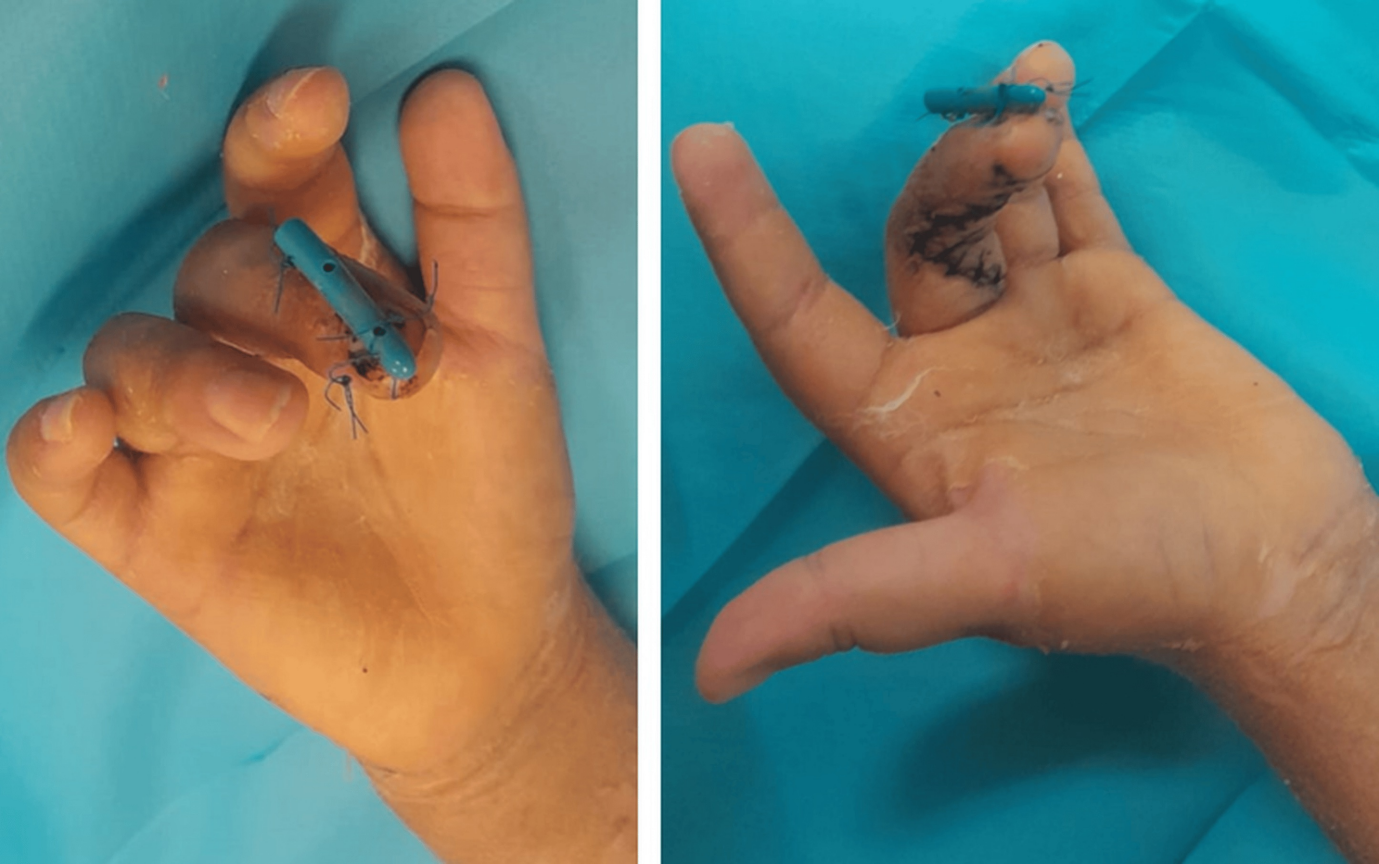
Clinical images at six months postoperatively Adequate flexion–extension mobility of the finger is observed.

**Figure 5:**
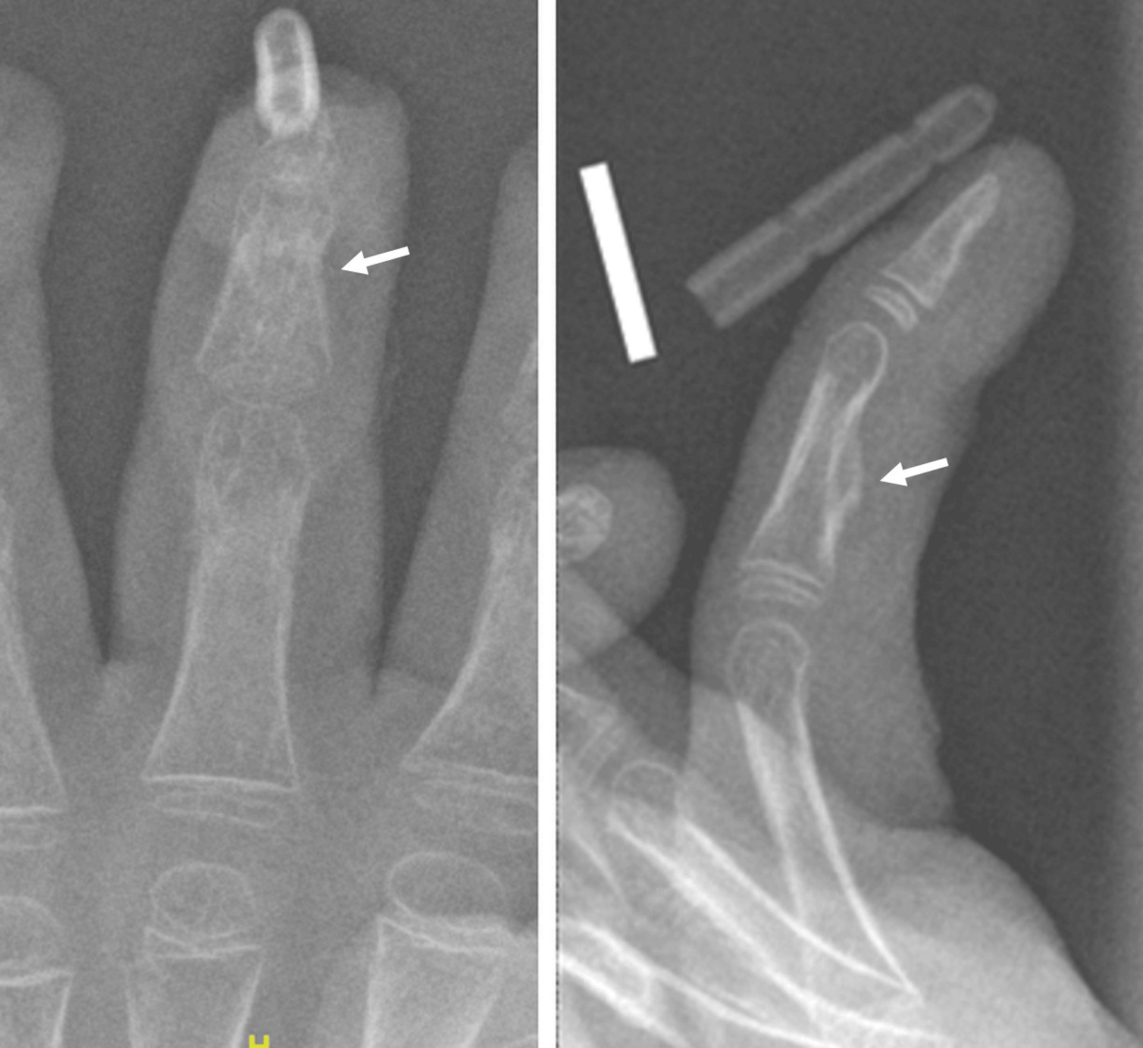
Consolidation of the initial ossification observed on follow-up radiographs obtained six weeks after surgery Left: anteroposterior view; Right: lateral view.

At the six-week follow-up, immobilization was discontinued and replaced with a protective forearm splint extending to the fingers for an additional two weeks. Flexion of the involved finger was possible (Figure 4), and control radiographs showed consolidation of the initial ossification, suggestive of periosteal injury secondary to a fracture of the middle phalanx at the time of the initial trauma (Figure 5). At eight weeks postoperatively, the protective splint and pull-out button were removed. Although a full range of motion at both interphalangeal joints had not yet been achieved, the patient was referred to occupational therapy.

## Discussion

Flexor tendon injuries of the hand account for approximately 1% of all hand injuries and have a significant impact on both patient function and societal burden [16]. In pediatric patients, these lesions are relatively uncommon, with an estimated annual incidence of 3.6 cases per 100,000 children, as reported by Cooper et al. [17]. Despite their rarity, diagnostic accuracy in this age group remains challenging. The inherently limited cooperation and communication skills of young children often hinder a complete examination, predisposing them to missed diagnoses and delayed recognition of tendon damage. Such delays can result in healing in a nonfunctional position, secondary loss of flexor function, or joint contracture secondary to volar plate injury [1]. These considerations emphasize the critical need for systematic and, when required, sedated assessment of pediatric hand injuries.

Therapeutic strategies are guided by the timing of diagnosis and tissue condition, ranging from primary repair in acute settings to delayed primary repair or reconstructive procedures in subacute or chronic cases. Acute repair may be contraindicated in the presence of comorbidities, infection, or significant soft-tissue loss [17]. The choice of technique must therefore be individualized, balancing surgical feasibility and functional recovery potential.

From an anatomical perspective, the Verdan classification remains essential to surgical planning. Zone two lesions-within the digital canal containing both the flexor digitorum superficialis and profundus tendons-pose the greatest technical challenge due to their limited vascularization and confined space, which favors adhesions [18]. In contrast, zone one injuries, which involve only the flexor digitorum profundus distal to the insertion of the superficialis, generally carry a better prognosis, as the distal interphalangeal joint contributes less to overall digital motion. Treatment options for zone one injuries include pull-out or micro-anchor fixation, both of which achieve functional reinsertion [18].

In our case, the initial skin laceration was located at the middle third of the middle phalanx of the third finger-clinically consistent with a potential zone two injury according to Verdan. However, intraoperative exploration revealed that the rupture was localized to zone one, underscoring the value of direct visualization when imaging and clinical findings are inconclusive. This finding supported the decision to perform a simple pull-out repair, which was deemed optimal based on tendon viability, minimal gap, and preserved soft-tissue coverage.

Postoperative management also reflects a balance between protection and mobilization. Evidence from adult series supports early active mobilization to minimize adhesions without increasing rupture risk. Although pediatric protocols remain debated, studies such as Cooper et al. [17] and others [19] suggest that early motion can be similarly beneficial in children. Their superior healing potential-attributable to enhanced tendon vascularity, greater scar remodeling capacity, and growth-related tissue adaptation-may facilitate spontaneous resolution of adhesions and better functional outcomes compared with adults [17].

## Conclusions

Early repair and mobilization are the preferred treatments for pediatric flexor tendon injuries, as in adults. However, these injuries are often underdiagnosed in children due to communication challenges, making a careful and systematic examination of volar finger wounds essential. The distinctive anatomical and physiological characteristics of children, including an active periosteum and rich vascularity, contribute to their generally favorable prognosis.

This case illustrates that even when diagnosis is delayed, good functional outcomes can be achieved with appropriate surgical management and rehabilitation. Nonetheless, the lack of standardized pediatric protocols and limited objective data remains an important challenge. Ongoing clinical vigilance and further research are needed to improve early recognition and evidence-based management of these injuries.
